# Validation of the rates of adverse event incidence in administrative healthcare data through patient chart review: A scoping review protocol

**DOI:** 10.12688/hrbopenres.13706.2

**Published:** 2024-12-12

**Authors:** Anna Connolly, Marcia Kirwan, Anne Matthews

**Affiliations:** 1 School of Nursing, Psychotherapy and Community Health, Dublin City University, Dublin, Leinster, Ireland

**Keywords:** Administrative healthcare data, Patient safety, Adverse events, Validation, Chart review

## Abstract

**Background:**

Patient safety is a key issue for health systems and a growing global public health challenge. Administrative healthcare data provide a coded summary of a patient and their encounter with the healthcare system. These aggregated datasets are often used to inform research and decisions relating to health service planning and therefore it is vital that they are accurate and reliable. Given the reported inaccuracy of these datasets for detecting and recording adverse events, there have been calls for validation studies to explore their reliability and investigate further their potential to inform research and health policy. Researchers have since carried out validation studies on the rates of adverse events in administrative data through chart reviews therefore, it seems appropriate to identify and chart the evidence and results of these studies within a scoping review.

**Methods:**

The scoping review will be conducted in accordance with the Joanna Briggs Institute (JBI) methodology for scoping reviews. A search of databases such as PubMed, CINAHL, ScienceDirect and Scopus will be conducted in addition to a search of the reference lists of sourced publications and a search for grey literature. Following this, Covidence will be used to screen the sourced publications and subsequently extract data from the included sources. A numerical summary of the literature will be presented in addition to a charting based on the qualitative content analysis of the studies included.

**Conclusions:**

This protocol provides the structure for the conduct of a review to identify and chart the evidence on validation studies on rates of adverse events in administrative healthcare data. This review will aim to identify research gaps, chart the evidence of and highlight any flaws within administrative datasets to improve extraction and coding practices and enable researchers and policy makers to use these data to their full potential.

## Introduction

Patient safety is a key issue for health systems and a growing global public health challenge. The human cost of patient safety incidents is high with adverse events causing injury, disability or death and resulting in suffering for patients and family members. The financial and economic burdens of patient safety incidents are also of concern with further healthcare expenditure required for increased length of stays in hospital and additional tests, treatments and healthcare to be provided to patients ( WHO, 2021b). The Harvard Medical Practice Study ( Brennan *et al.*, 1991) and the Institute of Medicine (2000) identified high rates of adverse events in hospital admissions and marked the beginning of the patient safety movement. This led to rates of adverse events becoming an important quality indicator across health systems. Adverse events have since been reported and measured however, challenges associated with the accuracy of their recording in administrative data has been widely acknowledged ( Kim *et al.*, 2022; Rodrigo-Rincon *et al.*, 2015). Evidently, patient safety is a key healthcare quality concern and with adverse events as main contributors to patient harm, their monitoring and measurement is a major priority.

Routinely collected healthcare data are regularly collected for purposes other than research ( Welk & Kwong, 2017). These data, frequently called administrative data, capture the patient’s characteristics, their diagnoses, treatments and procedures and, essentially provide a coded summary of the patient and their encounter with the healthcare system ( Healthcare Pricing Office, 2020).

The key events of a patient’s stay in hospital are documented in a discharge summary within their clinical chart or in their electronic health record (EHR). Once a patient has been discharged from hospital, their chart is translated into alphanumerical data by professional coding specialists ( Tang *et al.*, 2017). In addition to a patient’s discharge summary, clinical coders may also code information in documents such as nursing notes, consultation reports, progress notes, operative reports, pre- and post-operative reports and pathology reports ( Healthcare Pricing Office, 2020). Nursing notes have previously been identified as important sources of data for clinical coders ( Alonso *et al.*, 2020; Doktorchik *et al.*, 2020; Lucyk *et al.*, 2017) and are particularly insightful in relation to identifying adverse events such as pressure injuries, which are a well-recognised quality indicator ( Weller *et al.*, 2022). The data captured by the alphanumerical codes include patient diagnoses, procedures, hospital services used and any complications that arose during a patient’s stay.

Various classification systems such as the International Classification of Diseases (ICD) are used to code this data. The ICD translates diagnoses and other health-related issues from words into alphanumerical data, allowing for an efficient method of storing, retrieving, and analysing data ( WHO, 2016). Coding systems such as ICD allow for a consistent and standard way of recording, reporting and monitoring diseases. ICD is the is used by all members states of WHO, therefore data can be shared, understood and compared across hospitals, regions and countries ( WHO, 2023a). Various derived classifications or extensions of the fundamental ICD classifications have been developed and are used to monitor diagnoses in specific areas or settings ( WHO, 2023b). Clinical coders follow clearly defined ICD coding guidelines to assign alphanumeric codes to each diagnosis or event recorded in a patient’s chart ( O’Malley *et al.*, 2005). As the most widely used classification of diseases, ICD has had 10 iterations since 1900 ( O’Malley *et al.*, 2005). The 10 ^th^ revision of the ICD (ICD-10), which contains more than 155,000 codes ( DiSantostefano, 2009), has been widely used since its development about 30 years ago ( WHO, 2021a). An 11 ^th^ revision of the ICD (ICD-11 was adopted by World Health Assembly in 2019 and came into effect in 2022 ( WHO, 2023c). Various other coding systems such as the Current Procedural Terminology (CPT), which was developed by the American Medical Association, are used to capture and assign codes to aspects of healthcare data ( Dotson, 2013) however, ICD is recognised globally with all WHO member states committed to using this classification system.

Administrative data are often used at national and international levels in health service planning and to inform policy and epidemiological, clinical and biomedical research ( Hemkens *et al.*, 2016). They have also been utilised for carrying out audits, developing financial strategies and determining the distribution of health resources ( Burns *et al.*, 2012). Their use in the evaluation of the quality of health care delivery has also been well-recognised ( Clarke *et al.*, 2019). Given that these datasets are often used for purposes other than those for which they were initially collected, their use in research and evaluation has been labelled as a secondary use ( Jorm, 2015). Administrative data can contain rich and valuable information and their use in research has been increasingly recognised. These databases are advantageous for research as they allow research questions that require large sample sizes to be answered ( Harron *et al.*, 2017). Their potential to inform research to enhance the efficiency of health systems and improve population health is well-documented and has led to their increased use in research ( Jorm, 2015; Mitchell & Braithwaite, 2021; Moorthie *et al.*, 2022). Patient safety researchers in particular have been increasingly recognising the potential of using administrative data for research purposes and for the monitoring and reporting of patient safety events ( Raleigh *et al.*, 2008). Given the association between lower registered nurse staffing levels, increased mortality rates and other adverse outcomes ( Griffiths *et al.*, 2016), validating administrative datasets and ensuring their accuracy may also be beneficial in encouraging the potential for these datasets to be linked to nurse staffing data to demonstrate the impact of nurse staffing on patient outcomes and highlight areas for improvement.

Given that these data are used to inform research that may help to improve patients’ health and well-being and inform decisions that will impact on the quality of care that patients receive, it is vital that these administrative datasets are of high-quality and that precise and accurate diagnostic codes are used to generate them ( Gologorsky *et al.*, 2014; Ibrahim *et al.*, 2021). Accurate measurement of adverse events is central to improving patient safety and healthcare quality by allowing priority areas to be identified, improvement strategies to be designed and implemented interventions to be evaluated ( Classen *et al.*, 2011). Accurate data on adverse events may contribute to the development, implementation and evaluation of more targeted and evidence-based patient safety interventions that can prevent adverse events and improve quality of care ( Dillner *et al.*, 2023). Although these datasets can provide rich and detailed information, they are subject to limitations. Previous studies indicate that administrative datasets are not always accurate and therefore, may contribute to inaccuracies in research and lead to poorly informed decisions ( McGuckin *et al.*, 2022; Nicholls *et al.*, 2017). In particular, the inaccuracy of rates of adverse events in administrative data has been previously identified ( Kim *et al.*, 2022). Coding of primary diagnoses and procedures in administrative data is generally accurate however, coding of secondary diagnoses is less complete and therefore, adverse events rates are often underestimated ( Raleigh *et al.*, 2008).

Various challenges associated with using administrative data in research have been reported. Such challenges include not all data being abstracted from the patient chart, coded and represented in the database, incomplete data, difficulty determining the number of out-patient visits due to imprecise coding and difficulty capturing all relevant tests due to various diagnostic codes being used for the same test ( McGuckin *et al.*, 2022). The inconsistency in the definitions and terminology used in practice have previously been identified by Naessens *et al.* (2009) as another challenge to the accurate reporting of patient safety issues. Given that these administrative datasets are generated for purposes other than research, their accuracy and appropriateness for detecting adverse events is varied and their poor representation in some coding schemes has also previously been reported ( Bates *et al.*, 2003). The limited accuracy and variability of rates of hospital acquired infections in administrative data has also been identified as a challenge associated with using these datasets for research or benchmarking purposes. This has also resulted in calls for validation of these datasets and improvement of the algorithms used to generate them ( van Mourik *et al.*, 2015). Although the inaccuracy of administrative data for recording rates of adverse events has been previously reported by researchers such as Walther *et al.* (2021), who identified poor recording of postpartum haemorrhage within administrative data, the potential of these datasets to provide accurate information is also being recognised. Ackroyd-Stolarz *et al.* (2014) found a relatively high degree of accuracy in such datasets for identifying adverse events in older patients, therefore demonstrating the potential value of these datasets to inform research and improve patient safety.

Given the lack of consistency in the accuracy of these datasets for recording adverse events, there have been calls for validation studies to explore their reliability and investigate further their potential to inform research and health policy ( Ehrenstein *et al.*, 2016; Jorm, 2015; Nicholls *et al.*, 2017). Again, the potential of these datasets to inform research and policy in relation to patient safety and the human and economic cost of adverse events is evident, however specific calls for validating rates of adverse events within these datasets have been made ( Raleigh *et al.*, 2008; van Mourik *et al.*, 2015). Many researchers have since carried out validation studies on various administrative datasets therefore, it seems necessary to chart the evidence and results of these studies within this scoping review.

Validating administrative datasets is vital in determining the reliability and credibility of research based on this data and a common way of validating such data by manually comparing the coded data to the data recorded in a patient’s healthcare record ( Nissen *et al.*, 2019). Patient charts have been referred to as the ‘gold standard’ reference point in many validation studies ( Ehrenstein *et al.*, 2016), therefore this scoping review aims to present an overview of how this method of validating administrative data has been used by previous researchers. Various methodological approaches such as the Harvard Medical Practice Study (HMPS) and the Global Trigger Tool (GTT) can be used to conduct chart reviews. The HMPS method is a two-stage approach involving a nurse review for the triggering of charts, followed by a physician review of triggered charts to confirm rates of patient safety incidents using set definitions ( Brennan *et al.*, 1991) The GTT was developed by the Institute for Healthcare Improvement in response to demands for a less labour-intensive approach to chart reviewing. This two-stage approach involves a 20-minute screening phase for a comprehensive list of triggers, followed a physician review of the triggers using broader definitions to confirm the occurrence of an adverse event ( Rafter *et al.*, 2015). The HMPS method is a two-stage approach involving a nurse review for the triggering of charts, followed by a physician review of triggered charts to confirm rates of patient safety incidents using set definitions ( Brennan *et al.*, 1991) The GTT was developed by the Institute for Healthcare Improvement in response to demands for a less labour-intensive approach to chart reviewing. This two-stage approach involves a 20-minute screening phase for a comprehensive list of triggers, followed a physician review of the triggers using broader definitions to confirm the occurrence of an adverse event ( Rafter *et al.*, 2015).

### Inclusion criteria

In line with the JBI methodological framework for carrying out scoping reviews ( Peters *et al.*, 2020), the inclusion and exclusion criteria for studies that may potentially be included in the review will be defined by the population, concept and context screening criteria ( Table 1).

**Table 1.** Inclusion and exclusion criteria.

	Inclusion Criteria	Exclusion Criteria
**Population**	Sources that report on data derived from hospital-based patients.	Sources that report on data relating to any other population than hospital-based patients.
**Concept**	Sources that report on the validation of rates of adverse events in administrative data through reviews of patient charts.	Sources that do not primarily focus on the validation of rates of adverse events in administrative data through chart reviewing.
**Context**	Validation studies that have been conducted in hospital- based settings. There will be no limits placed on geographical location of where sources of evidence have been published.	Studies that report on validating data in any other setting than hospital-based settings.
**Types of Evidence** **Sources**	Full text peer-reviewed and non-peer reviewed sources ( *e.g.,* grey literature).	Duplications and sources of evidence that cannot be made available in full text.
**Language**	Sources available in English.	Sources published in languages other than English.
**Time period**	Sources of evidence published between 1991 and 2023.	Sources of evidence published before 1991.

### Population

As this is a protocol for a scoping review exploring the use of chart reviews to validate rates of adverse events in administrative data, the population of interest is hospital-based patients. These are Patients who have been discharged from hospital and have had the information within their medical chart extracted, assigned ICD codes, and recorded in administrative data Hospital-based patients’ data will have been used to inform the publications that will be included in this scoping review, therefore making them the population of interest in this review. This research will seek to focus on identifying and charting the evidence of reviewing hospital-based patient’s charts as a method of carrying out validation studies on rates of adverse events in administrative datasets.

### Concept

Validating the accuracy of the coding of administrative data is key for ensuring that the policy, practice and research informed by such datasets is credible and robust ( Nicholls *et al.*, 2017). A review of patient charts is a common and effective way of validating the accuracy of administrative data as it allows the coded data recorded in the administrative dataset to be compared to the data maintained within hospital notes or discharge summaries within patient charts ( Burns *et al.*, 2012). The chart review methodology is an efficient way of determining whether the administrative data is an accurate reflection of the patient’s physical medical chart ( Nissen *et al.*, 2019). The concept explored in this scoping review will be the validation of rates of adverse events in administrative data through chart review.

### Context

Validation studies that have been conducted in hospital-based settings will be considered for inclusion in this review. Chart reviews that have been conducted as part of a validation of rates of adverse events in administrative datasets that have been carried out in any country will be included in this review. There will be no limitation on the context of the administrative data in order to allow a comprehensive review to be carried out.

### Types of evidence sources

The types of sources of evidence to be included in this review will be left open to allow a comprehensive review of all available literature to be carried out. This will allow for the inclusion of various types of publications and therefore, should provide an in-depth presentation of the previous literature published in relation to using chart reviews to validate administrative data.

## Methods

This scoping review will be conducted in accordance with the Joanna Briggs Institute (JBI) methodology for scoping review ( Peters *et al.*, 2020). The JBI methodological framework will be used to guide this scoping review and ensure that the review is carried out appropriately.

### Search strategy

The sources of evidence that will be included in this scoping review will be identified and collated using a three-step process as outlined by the JBI ( Peters *et al.*, 2020). The first step of this process will involve an initial search of two databases. The databases included in this initial search will be PubMed (MEDLINE) and CINAHL. Following this preliminary search, an analysis of the text words in the titles and abstracts of identified papers will be carried out. The indexing terms used to describe the retrieved papers will also be analysed. The second step of this process will involve using the identified keywords and index terms to perform a second search of all included databases. The databases included in this step will include PubMed (MEDLINE), CINAHL, Web of Science and Scopus. The third and final step of this process will involve a search of the reference lists of identified publications for additional sources of materials to be included in this scoping review. The reference lists of all publications selected from full text or those included in the review will be searched in order to ensure a comprehensive and in-depth search for existing literature. A search for grey literature will also be conducted to ensure that the review is as thorough as possible ( Arksey & O’ Malley, 2005). Grey literature refers to literature that is not commercially published and not usually available within standard databases. Examples of such literature include government reports, conference proceedings, theses and dissertations. A search for this type of literature is beneficial as many researchers publish through other means, such as those mentioned above, and therefore, this allows for the inclusion of a wider scope of material. Also, although such materials may not be included in the final scoping review, they can aid in identifying data sources, research currently being undertaken in the area or complete study reports ( Pawliuk *et al.*, 2021).

Sources included in this scoping review will be limited to publications written in English as it will not be feasible for the research team to translate publications published in other languages into English. Literature published over the past 30 years, between 1991 and 2023, will be eligible for inclusion in this study. This time period has been chosen as the patient safety movement was instigated by Brennan *et al.* (1991) about 30 years ago following their study that investigated the incidence of adverse events and negligence in hospitalised patients through the use of chart reviews.

A librarian from Dublin City University has been consulted and their advice was sought in relation to developing a sample search strategy ( Table 2). Advice from the librarian will continue to be sought in relation to the search strategy to ensure that a comprehensive search of the available literature is completed. The research team will continue to work closely with the librarian throughout the data collection phase of the scoping review to make certain that repetitions of the search strategy result in all key and relevant literature being gathered.

**Table 2.** Sample search strategy.

Search	Search Terms
**S1**	“discharge data” OR "hospital discharge data" OR “routinely collected data” OR "routinely collected discharge data" OR “administrative data” OR "administrative health data" OR "healthcare administrative data"
**S2**	verif* OR valid* OR compar* OR evaluat*
**S3**	S1 AND S2
**S4**	"chart review" OR "record review" OR "medical record review" OR "clinical notes" OR "retrospective chart review"
**S5**	S3 AND S4
**S6**	"adverse event*" OR "adverse outcome*" OR "healthcare acquired complication*" OR "patient safety incident*"
**S7**	S5 AND S6

### Study selection

After the search for literature to be potentially included in this scoping review, all identified publications will be gathered and their citations will be stored in the bibliographic software Zotero.

The review management tool Covidence will be used to screen the collected publications and subsequently extract data from included sources. The references of the sourced publications will be imported to Covidence from Zotero and any duplicates will be automatically identified and removed. Using Covidence, two members of the research team will independently screen each of the imported studies for inclusion based on title and abstract examination. The researchers will individually assess the eligibility of each publication for inclusion in the review by making reference to the inclusion criteria. The researchers will each have the option of selecting yes, no or maybe at this stage of the selection process. Any conflicts that arise between the two researchers at this initial screening stage will be resolved by discussion and by consulting a third member of the research team to reach a final decision if necessary. Sources of data that received a yes vote from both researchers and are deemed potentially relevant based on the initial assessments made by researchers after examining the titles and abstracts will be retrieved in their full-text version. The full-text versions of potential studies for inclusion will be read and again, be assessed against the inclusion criteria by two researchers independently. The researchers will have the option in Covidence to include or exclude publications at this stage. If the researchers vote to exclude any publication, they will be prompted to select a reason for exclusion. This decision-making process will be documented and reported in the scoping review and also displayed in a PRISMA flowchart. The researchers will then be guided to the consensus process. The consensus screen in Covidence highlights the number of conflicts between the two researchers and allows a final decision to be reached in relation to the inclusion of publications. In the event that any disagreement between the two members of the research team assessing the studies for inclusion in the scoping review arises, a third member of the research team will be consulted, and their input will be used to resolve such conflicts of opinions. In line with the JBI guidelines ( Peters *et al.*, 2020), a PRISMA flowchart of the review process will be developed and included in the review. This flowchart will detail the full flow of the review process from the initial search to the presentation of included sources.

### Data extraction

The data extraction method for this scoping review will firstly involve the development of a data extraction template. As previously mentioned, the data will be extracted from the publications selected for inclusion in the scoping review within Covidence. The key information that will be extracted will include the following: name of author(s), year of publication, geographical location of study, aim of the study, methodology and key findings that relate to the research question. The data extraction method will be piloted and trialled on two or three sources to ensure that all relevant data is extracted. After this step, the extraction template will be updated to ensure that it is an appropriate method of collecting all necessary data. The piloting of the template will be carried out by two members of the research team as recommended by Peters *et al.* (2020). Once these data have been extracted, they will be exported to Microsoft Excel.

### Data analysis and presentation

In line with the JBI methodological framework for scoping reviews, the extracted data will be arranged into a charting table in order to provide the reviewers and readers with a clear and descriptive summary of the publications included in the scoping review ( Peters *et al.*, 2020). According to Peters *et al.* (2020), the data charting can be an iterative process and therefore, the charting tool will be piloted, and the table will be continuously updated. The results of this scoping review will be mapped descriptively in order to answer the research question. A narrative account of the included literature will be presented. A numerical summary of the literature will be presented in addition to a qualitative content analysis of the studies included in the scoping review. Both the numerical summary and qualitative content analysis will be of a descriptive nature as the aim of a scoping review is to present an overview of the literature reviewed rather than an assessment of the quality of the included materials ( Arksey & O’Malley, 2005; Peters *et al.*, 2020). The descriptive numerical summary will include information such as the overall number of publications that have been included, the years of publication, the geographical location where the studies were conducted, and the types of study designs used. The qualitative content analysis will answer the research question by providing a description of the results of each of the included studies. The data extracted from the included studies will be presented in a tabular and descriptive format.

Considering the implications of the findings produced by a scoping review has been highlighted as an important part of the scoping review methodological framework ( Levac *et al.*, 2010). Given that this is viewed as an essential element of conducting a scoping review, the implications of the results of this scoping review will be considered in relation to the identification of research gaps or calls for further research in the area.

### Study status

The scoping review is currently in the preliminary stages of searching the databases. Step 1 of the three-step search strategy, as outlined by Peters *et al.* (2020) for identifying and collating sources of evidence to inform the scoping review, is currently underway. An initial search of databases for potentially relevant sources of evidence is being performed.

## Conclusions

This protocol provides the structure for the conduction of a review to identify and chart the evidence of validation studies of rates of adverse events in administrative data. In line with the JBI guidelines ( Peters *et al.*, 2020), any deviations of the scoping review from the protocol will be acknowledged and discussed in the scoping review. It is widely acknowledged that administrative data have the potential to enhance patient safety and be used as a quality indicator however, given that the rates of adverse events in these datasets have been frequently reported as inaccurate, there is a strong rationale to conduct this scoping review. This review will aim to present an overview of the chart review approaches and tools that have been used to validate rates of AEs in administrative datasets and ultimately highlighting the need to develop a systematic approach to measuring rates of AEs in administrative data. This will allow the accuracy of these datasets to be improved, which will enable researchers and policy makers to use them to their full potential to improve patient safety and healthcare quality.

## Data Availability

No data are associated with this article.

## References

[ref-1] Ackroyd-Stolarz S Bowles SK Giffin L : Validating administrative data for the detection of adverse events in older hospitalized patients. *Drug Healthc Patient Saf.* 2014;6:101–108. 10.2147/DHPS.S64359 25143755 PMC4137915

[ref-2] Alonso V Santos J Pinto M : Health Records as the basis of clinical coding: Is the quality adequate? A qualitative study of medical coders’ perceptions. *Health Inf Manag.* 2020;49(1):28–37. 10.1177/1833358319826351 30744403

[ref-3] Arksey H O'Malley L : Scoping studies: towards a methodological framework. *Int J Soc Res Methodol.* 2005;8(1):19–32. 10.1080/1364557032000119616

[ref-4] Bates DW Evans RS Murff H : Detecting adverse events using information technology. *J Am Med Inform Assoc.* 2003;10(2):115–128. 10.1197/jamia.m1074 12595401 PMC150365

[ref-5] Brennan TA Leape LL Laird NM : Incidence of adverse events and negligence in hospitalized patients. Results of the Harvard Medical Practice Study I. *N Engl J Med.* 1991;324(6):370–376. 10.1056/NEJM199102073240604 1987460

[ref-6] Burns EM Rigby E Mamidanna R : Systematic review of discharge coding accuracy. *J Public Health (Oxf).* 2012;34(1):138–148. 10.1093/pubmed/fdr054 21795302 PMC3285117

[ref-7] Clarke GM Conti S Wolters AT : Evaluating the impact of healthcare interventions using routine data. *BMJ.* 2019;365: l2239. 10.1136/bmj.l2239 31221675 PMC6584784

[ref-8] Classen DC Resar R Griffin F : ‘Global Trigger Tool’ shows that adverse events in hospitals may be ten times greater than previously measured. *Health Aff (Millwood).* 2011;30(4):581–589. 10.1377/hlthaff.2011.0190 21471476

[ref-9] Dillner P Eggenschwiler LC Rutjes AW : Incidence and characteristics of adverse events in paediatric inpatient care: a systematic review and meta-analysis. *BMJ Qual Saf.* 2023;32(3):133–149. 10.1136/bmjqs-2022-015298 36572528 PMC9985739

[ref-10] DiSantostefano J : International Classification of Diseases 10th Revision (ICD-10). *J Nurse Pract.* 2009;5(1):56–57. 10.1016/j.nurpra.2008.09.020

[ref-11] Doktorchik C Lu M Quan H : A qualitative evaluation of clinically coded data quality from health information manager perspectives. *Health Inf Manag.* 2020;49(1):19–27. 10.1177/1833358319855031 31284769

[ref-12] Dotson P : CPT ^®^ Codes: What are they, why are they necessary, and how are they developed? *Adv Wound Care (New Rochelle).* 2013;2(10):583–587. 10.1089/wound.2013.0483 24761332 PMC3865623

[ref-13] Ehrenstein V Petersen I Smeeth L : Helping everyone do better: a call for validation studies of routinely recorded health data. *Clin Epidemiol.* 2016;8:49–51. 10.2147/CLEP.S104448 27110139 PMC4835131

[ref-14] Gologorsky Y Knightly JJ Lu Y : Improving discharge data fidelity for use in large administrative databases. *Neurosurg Focus.* 2014;36(6):E2. 10.3171/2014.3.FOCUS1459 24881634

[ref-50] Griffiths P Ball J Drennan J : Nurse staffing and patient outcomes: strengths and limitations of the evidence to inform policy and practice. A review and discussion paper based on evidence reviewed for the National Institute for Health and Care Excellence Safe Staffing guideline development. *Int J Nurs Stud.* 2016;63:213–225. 10.1016/j.ijnurstu.2016.03.012 27130150

[ref-15] Harron K Dibben C Boyd J : Challenges in administrative data linkage for research. *Big Data Soc.* 2017;4(2): 2053951717745678. 10.1177/2053951717745678 30381794 PMC6187070

[ref-16] Healthcare Pricing Office: Clinical Coding.2020; (Accessed 12 January 2023). Reference Source

[ref-17] Hemkens LG Contopoulos-Ioannidis DG Ioannidis JPA : Routinely collected data and comparative effectiveness evidence: promises and limitations. *CMAJ.* 2016;188(8):E158–E164. 10.1503/cmaj.150653 26883316 PMC4868623

[ref-18] Ibrahim H Liu X Zariffa N : Health data poverty: an assailable barrier to equitable digital health care. *Lancet Digit Health.* 2021;3(4):e260–e265. 10.1016/S2589-7500(20)30317-4 33678589

[ref-19] Institute of Medicine: To Err Is Human: Building a Safer Health System. Washington, DC: The National Academies Press,2000. 10.17226/9728 25077248

[ref-20] Jorm L : Routinely collected data as a strategic resource for research: priorities for methods and workforce. *Public Health Res Pract.* 2015;25(4): e2541540. 10.17061/phrp2541540 26536502

[ref-21] Kim J Choi EY Lee W : Feasibility of Capturing Adverse Events From Insurance Claims Data Using *International Classification of Diseases, Tenth Revision*, Codes Coupled to Present on Admission Indicators. *J Patient Saf.* 2022;18(5):404–409. 10.1097/PTS.0000000000000932 35948289 PMC9329045

[ref-22] Levac D Colquhuon H O’ Brien KK : Scoping studies: advancing the methodology. *Implement Sci.* 2010;5:69. 10.1186/1748-5908-5-69 20854677 PMC2954944

[ref-23] Lucyk K Tang K Quan H : Barriers to data quality resulting from the process of coding health information to administrative data: a qualitative study. *BMC Health Serv Res.* 2017;17(1):766. 10.1186/s12913-017-2697-y 29166905 PMC5700659

[ref-24] McGuckin T Crick K Myroniuk TW : Understanding challenges of using routinely collected health data to address clinical care gaps: a case study in Alberta, Canada. *BMJ Open Qual.* 2022;11(1): e001491. 10.1136/bmjoq-2021-001491 34996811 PMC8744094

[ref-25] Mitchell R Braithwaite J : Evidence-informed health care policy and practice: using record linkage to uncover new knowledge. *J Health Serv Res Policy.* 2021;26(1):62–67. 10.1177/1355819620919793 32326762

[ref-26] Moorthie S Hayat S Zhang Y : Rapid systematic review to identify key barriers to access, linkage, and use of local authority administrative data for population health research, practice, and policy in the United Kingdom. *BMC Public Health.* 2022;22(1):1263. 10.1186/s12889-022-13187-9 35764951 PMC9241330

[ref-27] Naessens JM Campbell CR Huddleston JM : A comparison of hospital adverse events identified by three widely used detection methods. *Int J Qual Health Care.* 2009;21(4):301–307. 10.1093/intqhc/mzp027 19617381

[ref-28] Nicholls SG Langan SM Benchimol EI : Routinely collected data: the importance of high-quality diagnostic coding to research. *CMAJ.* 2017;189(33):E1054–E1055. 10.1503/cmaj.170807 28827435 PMC5566604

[ref-29] Nissen F Quint JK Morales DR : How to validate a diagnosis recorded in Electronic Health Records. *Breathe (Sheff).* 2019;15(1):64–68. 10.1183/20734735.0344-2018 30838062 PMC6395976

[ref-30] O'Malley KJ Cook KF Price MD : Measuring diagnoses: ICD code accuracy. *Health Serv Res.* 2005;40(5 Pt 2):1620–1639. 10.1111/j.1475-6773.2005.00444.x 16178999 PMC1361216

[ref-31] Pawliuk C Brown HL Widger K : Optimising the process for conducting scoping reviews. *BMJ Evid Based Med.* 2021;26(6):312–316. 10.1136/bmjebm-2020-111452 33087454

[ref-32] Peters MDJ Godfrey C McInerney P : Chapter 11: Scoping Reviews. In: Aromataris, E. and Munn, Z. (eds.) *JBI Manual for Evidence Synthesis*. JBI, 2020.2020. 10.46658/JBIMES-20-12

[ref-33] Rafter N Hickey A Condell S : Adverse events in healthcare: learning from mistakes. *QJM.* 2015;108(4):273–277. 10.1093/qjmed/hcu145 25078411

[ref-34] Raleigh VS Cooper J Bremner SA : Patient safety indicators for England from hospital administrative data: case-control analysis and comparison with US data. *BMJ.* 2008;337: a1702. 10.1136/bmj.a1702 18930971 PMC2569150

[ref-35] Rodrigo-Rincon I Martin-Vizcaino MP Tirapu-Leon B : Validity of the clinical and administrative databases in detecting post-operative adverse events. *Int J Qual Health Care.* 2015;27(4):267–275. 10.1093/intqhc/mzv039 26082462

[ref-36] Tang KL Lucyk K Quan H : Coder perspectives on physician-related barriers to producing high-quality administrative data: a qualitative study. *CMAJ Open.* 2017;5(3):E617–E622. 10.9778/cmajo.20170036 28827414 PMC5621953

[ref-37] van Mourik MS van Duijn PJ Moons KG : Accuracy of administrative data for surveillance of healthcare-associated infections: a systematic review. *BMJ Open.* 2015;5(8): e008424. 10.1136/bmjopen-2015-008424 26316651 PMC4554897

[ref-38] Walther D Halfon P Tanzer R : Hospital discharge data is not accurate enough to monitor the incidence of postpartum hemorrhage. *PLoS One.* 2021;16(2): e0246119. 10.1371/journal.pone.0246119 33534862 PMC7857548

[ref-39] Welk B Kwong J : A review of routinely collected data studies in urology: methodological considerations, reporting quality, and future directions. *Can Urol Assoc J.* 2017;11(3–4):136–141. 10.5489/cuaj.4101 28515814 PMC5434499

[ref-40] Weller CD Turnour L Connelly E : Clinical coders’ perspectives on pressure injury coding in acute care services in Victoria, Australia. *Front Public Health.* 2022;10: 893482. 10.3389/fpubh.2022.893482 35719639 PMC9198603

[ref-41] WHO: International statistical classification of diseases and related health problems - 10 ^th^ Revision Volume 2 Instruction Manual. 5 ^th^edn. Geneva: World Health Organization,2016. Reference Source

[ref-42] WHO: Global Patient Safety Action Plan 2021-2030: towards eliminating avoidable harm in healthcare. Geneva: World Health Organization,2021b. Reference Source

[ref-43] WHO: Importance of ICD.2023a; (Accessed 1 February 2023). Reference Source

[ref-44] WHO: Other Classifications.2023b; (Accessed 1 February 2023). Reference Source

[ref-45] WHO: International Statistical Classification of Diseases and Related Health Problems (ICD).2023c; (Accessed 20 February 2023). Reference Source

[ref-46] World Health Organization (WHO): History of the development of ICD.2021a; (Accessed 1 February 2023). Reference Source

