# Supplementary material for: Validation of the rates of adverse event incidence in administrative healthcare data through patient chart review: A scoping review protocol
Source: HRB Open Res. 2024 Dec 12;6:21. Originally published 2023 Mar 23. [Version 2] doi: 10.12688/hrbopenres.13706.2 (PMC11808841; doi:10.12688/hrbopenres.13706.2)
Supplement: Peer review history

# Reviewer report: Reviewer response for version 2

**DOI:** 10.21956/hrbopenres.15420.r44294

**Published:** 2025-01-16

**Authors:** Dean F Sittig

**Affiliations:** 1 University of Texas Health Science Center at Houston, Houston, TX, USA

**Your Report**

 Overall

 Important scoping review about validating adverse event incidence through chart reviews. The introduction and conclusion need to be revised. The JBI methodology is followed correctly. The paper should be revised and resubmitted for further consideration. In general, someone, either the authors or the editors of the journal need to take responsibility for making sure that there a no errors in the manuscript. Currently, there are typos, duplications, and words that don’t make sense.

 Abstract

1. Background – clearly state objective of scoping review and scoping review question.
2. Methods – What is Covidence? Maybe mention a software was used and then elaborate the use of Covidence in the methods section in body of the manuscript.

Manuscript

1. Introduction/Background –
  1. Multiple typos (for example: in the 3 ^rd^ paragraph: patient’s stay needs a space in between, include “the” in front of hospital). A proofread is needed once more to check for grammar and spelling errors.
  2. Evidently?, patient safety is a key healthcare…remove evidently
  3. Para 2: administrative data contain some patient characteristics like age, gender, but are most often de-identified as much as possible. This should be mentioned.
  4. Mention ICD is available in multiple languages
  5. Weave in 2 ^nd^ paragraph into the 5 ^th^ paragraph to make it more concise.
  6. Overall introduction still contains repetition and can be condensed further.
  7. Clearly state objective of scoping review and scoping review question prior to introducing inclusion sub-heading.
2.  Pg 4 last para – The HMPS method is a two-stage is written 2x. this paper requires significant proof-reading before it can be published.
3. Pg 5: population section missing period at end of sentence ..administrative data Hospital-based…
  1. Population para is overly redundant and duplicative. Consider 1 good sentence to say you are focused on hospital patients.
4. Context para: first sentence should be removed. It is duplicative of concept section.
5. Types of evidence open? I think you should at least limit to published studies in English (unless you can read other languages) of adults (or will you include children, if so, say that). I think peer-reviewed literature seems like a reasonable limit at well.
6. Table 1 is not very useful. The exclusion criteria seem to be simply the opposite of the inclusion criteria. That is a given. I think the exclusion should be like: No children will be included in the study; no rehab hospitals’ data will be included, etc. Also, exclusion sources of evidence published after 2023, apparently. Which I would at least move to the end of 2024, now.
7. Conclusion –
  1. Citation is needed for “rates of AE have been frequently reported as inaccurate”.
  2. Need to mention similar or previous studies in this area to differentiate what this paper is adding to the field.
  3. Ethics approval or waiver statement is needed.

Is the study design appropriate for the research question?

Yes

Is the rationale for, and objectives of, the study clearly described?

Yes

Are sufficient details of the methods provided to allow replication by others?

Yes

Are the datasets clearly presented in a useable and accessible format?

Not applicable

Reviewer Expertise:

NA

I confirm that I have read this submission and believe that I have an appropriate level of expertise to confirm that it is of an acceptable scientific standard, however I have significant reservations, as outlined above.

# Reviewer report: Reviewer response for version 2

**DOI:** 10.21956/hrbopenres.15420.r44039

**Published:** 2024-12-28

**Authors:** Keith Marsolo

**Affiliations:** 1 Duke University School of Medicine, Durham, NC, USA

The authors have made sufficient revisions to address the concerns raised in my review.

Is the study design appropriate for the research question?

Partly

Is the rationale for, and objectives of, the study clearly described?

Partly

Are sufficient details of the methods provided to allow replication by others?

Yes

Are the datasets clearly presented in a useable and accessible format?

Not applicable

Reviewer Expertise:

informatics, real-world data research.

I confirm that I have read this submission and believe that I have an appropriate level of expertise to confirm that it is of an acceptable scientific standard.

# Reviewer report: Reviewer response for version 1

**DOI:** 10.21956/hrbopenres.14991.r36413

**Published:** 2023-11-14

**Authors:** Angela Flynn

**Affiliations:** 1 University College Cork, Cork, County Cork, Ireland

This is a very interesting protocol for a necessary scoping review. The authors have clearly identified the importance of the accuracy and validity of important healthcare incident data in the inpatient clinical setting. 

 I was uncertain as to what precisely was meant by "administrative healthcare data" and, as has been mentioned by another reviewer, I wondered whether there may be other sources to include - such as electronic health record. The universality of this data is very well explained however, while specific diagnoses are globally understood via the ICD classification the adverse incidents may not yet have such a universal understanding. This scoping review aims to work towards that goal. It may be worth emphasising further how the ultimate goal might be to in fact prevent and anticipate adverse incidents and improve quality of care. 

 You mention a number of potential benefits of this work but I wondered whether it might be worthwhile pointing out the capacity to link this data with staffing information to demonstrate the impact of safe/unsafe staffing. In the context of a global nursing and health professional shortage, it is a point worth making.

 In terms of other detail, the introduction seems a little too long. The sentence beginning "Whilst the inaccuracy of..." needs to be reworded. The detail around various methods of chart reviewing seems excessive.

 Population - the sentence "This population is of..." needs attention. 

 Concept - the last two sentences in this seem repetitive.

 Study Selection - this piece is good but you might just clarify in one line how many researchers will review each paper for the inclusion/exclusion decision - is it one or two?

 Otherwise an interesting protocol and I look forward to reading the review results.

Is the study design appropriate for the research question?

Yes

Is the rationale for, and objectives of, the study clearly described?

Yes

Are sufficient details of the methods provided to allow replication by others?

Yes

Are the datasets clearly presented in a useable and accessible format?

Not applicable

Reviewer Expertise:

Nursing, Health inequities, Inclusion Health

I confirm that I have read this submission and believe that I have an appropriate level of expertise to confirm that it is of an acceptable scientific standard.

# Response

**Published:** 2024-12-05

**Authors:** Anna Connolly

**Affiliations:** Dublin City University, Ireland

We would like to thank the reviewer for their support of our work and constructive feedback on our manuscript.

 We have amended the manuscript to provide further clarity on what is meant by administrative healthcare data and included a mention of electronic heath records as another source from which administrative data can be generated.

 We have also amended the manuscript to place further emphasis on the prevention of adverse events and the improvement of quality care as the ultimate goal of validating administrative datasets and ensuring their accuracy. Furthermore, the potential benefit of this work to highlight the capacity of linking accurate administrative data with staffing information to demonstrate the impact of safe/unsafe staffing has also been addressed in the revised manuscript.  

 On review of the manuscript, we have reduced the introduction section and provided a more concise overview of the chart review methodologies. Also, in light of the comments, we have amended the population and concept sections in the manuscript.

 Finally, the manuscript has also been amended to provide a clearer description of the number of researchers involved in the reviewing of the papers for inclusion or exclusion. Two researchers each individually reviewed papers for inclusion/exclusion.

 The scoping review has since been completed and published in the International Journal for Quality in Health Care and is available at: https://academic.oup.com/intqhc/article/36/2/mzae037/7658312?searchresult=1.

 Thank you again for your comments, feedback and support of this research.

# Reviewer report: Reviewer response for version 1

**DOI:** 10.21956/hrbopenres.14991.r36049

**Published:** 2023-09-27

**Authors:** Keith Marsolo

**Affiliations:** 1 Duke University School of Medicine, Durham, NC, USA

This protocol describes a scoping review related to adverse events obtained from administrative data, particularly related to methods of validation for adverse event detection. The methods are generally good, though the proposed search terms are likely to miss many potential publications, at least from the United States (US).

 The protocol makes frequent use of the term "administrative data," though that is not routinely used in many fields. In the US, for instance, "administrative claims data" is used to refer to information available from insurance companies or health plans. In contrast, data available from health systems is often simply called "electronic health record" or EHR data. This protocol is mostly about identifying articles that describe validation of adverse event detection method, which implies that the authors are interested in EHR data. However, the search terms may be more likely to bring back articles related to administrative claims. Claims data are often used in health services research, and US initiatives like FDA's Sentinel will often look for adverse events within these datasets, but those adverse events are not necessarily the same ones that occur within a hospital setting. I would recommend revisiting the search terms and include reference to EHR datasets.

 In adddition, there is a fair amount of redundant text in the Introduction (for instance, 2 ^nd^ paragraph and initial sentences of paragraphs 3 and 5). If this text is intended to be used in a subsequent publication, it should be condensed.

Is the study design appropriate for the research question?

Partly

Is the rationale for, and objectives of, the study clearly described?

Partly

Are sufficient details of the methods provided to allow replication by others?

Yes

Are the datasets clearly presented in a useable and accessible format?

Not applicable

Reviewer Expertise:

informatics, real-world data research.

I confirm that I have read this submission and believe that I have an appropriate level of expertise to confirm that it is of an acceptable scientific standard, however I have significant reservations, as outlined above.

# Response

**Published:** 2024-12-05

**Authors:** Anna Connolly

**Affiliations:** Dublin City University, Ireland

We would like to thank the reviewer for their support of our work and constructive feedback on our manuscript.

 We appreciate and have considered the feedback in relation to the inclusion of reference to electronic health records in the search strategy terms for the identification of publications for inclusion in this review. The manuscript has been amended to reflect the electronic health record as a source from which administrative data can be derived. This review intended to provide an overview of the chart review methods used to validate coded data such as those in administrative datasets rather than validating electronic health record data itself, hence why reference to EHR datasets alone was not included in the search terms. Furthermore, on preliminary searches using the search terms published in this protocol, many of the results pertained to studies that took place in the US therefore, we believe that the developed search strategy is sufficient for identifying relevant research articles.

 Also, the text in the opening section has been reduced to provide a more concise introduction to the protocol.

 The scoping review has since been completed and published in the International Journal for Quality in Health Care and is available at: https://academic.oup.com/intqhc/article/36/2/mzae037/7658312?searchresult=1.

 Thank you again for your comments, feedback and support of this research.
